# A Novel Vaccine Candidate: Recombinant *Toxoplasma gondii* Perforin-Like Protein 2 Stimulates Partial Protective Immunity Against Toxoplasmosis

**DOI:** 10.3389/fvets.2021.802250

**Published:** 2022-02-15

**Authors:** Xiaowei Tian, Hanqi Sun, Meng Wang, Guangmin Wan, Tong Xie, Xuefang Mei, Zhenchao Zhang, Xiangrui Li, Shuai Wang

**Affiliations:** ^1^Xinxiang Key Laboratory of Pathogenic Biology, Department of Pathogenic Biology, School of Basic Medical Sciences, Xinxiang Medical University, Xinxiang, China; ^2^MOE Joint International Research Laboratory of Animal Health and Food Safety, College of Veterinary Medicine, Nanjing Agricultural University, Nanjing, China

**Keywords:** *Toxoplasma gondii*, perforin-like protein 2, RH and PRU strains, vaccine, BALB/c mice, protective immunity

## Abstract

*Toxoplasma gondii* is an apicomplexan pathogen infecting 2 billion people and numerous livestock, causing a major threat to economies and human health. Passive-active immunoprophylaxis is an efficient approach to provide protection against toxoplasmosis. *T. gondii* perforin-like protein 2 (TgPLP2) contains a membrane attack complex/perforin (MACPF) domain, making it a potential vaccine candidate. Here, we aimed to assess the protection efficacy of TgPLP2 using Bagg albino/c (BALB/c) mice model. The *Escherichia coli* system was used to obtain the recombinant TgPLP2 (rTgPLP2). Mice challenged by anti-rTgPLP2 polyclonal antibodies (PcAb) pretreated tachyzoites showed obviously increased survival outcomes. In addition, mice that passively received anti-rTgPLP2 PcAb following a lethal dose of tachyzoites infection had longer survival time compared with phosphate-buffered saline (PBS) controls. Furthermore, we demonstrated that immunization with rTgPLP2 could prolong survival in RH strain infected mice and resulted in the lowest brain cysts size and number of Prugniaud (PRU) genotype II strain infected mice. High levels of *Toxoplasma*-specific IgG, IgG1, IgG2a, and cytokines (IFN-γ and IL-10) were produced after two immunizations with rTgPLP2. Together these results indicated that TgPLP2 can induce both humoral and cellular immune responses to protect host against infection and thus is a potential candidate for *T. gondii* vaccines.

## Introduction

Toxoplasmosis is an important zoonotic parasitic disease with worldwide distribution, caused by an obligate intracellular parasite *Toxoplasma gondii* (*T. gondii*) (1, 2). This disorder can lead to infantile encephalitis, miscarriage in pregnant women and livestock, and brain toxoplasmosis and even death of immunocompromised people (3–5), which is seriously harmful to human and animal health. As the current drug treatment cannot completely cure toxoplasmosis, immunoprophylaxis has become the first choice to control the disease (6–9).

Perforin is a secretory protein that contains a membrane attack complex/perforin (MACPF) domain, which functions to induce killing of infected cells and the invading pathogen by forming a pore on the target cell membrane (10). Recent researches suggested that the phylum Apicomplexa genome could encode different perforin-like proteins in different stages of the parasite life cycle, and some of them mainly serve important roles (11). The MACPF protein found in Apicomplexa parasites was the major protein inducing membrane perforation (12). *T. gondii* has two perforin-like proteins (TgPLP1 and TgPLP2) that contain MACPF domain.

Current studies suggest *T. gondii* perforin-like proteins (PLPs) may therefore serve as good vaccine candidates. TgPLP1 is a protective antigen against *T. gondii*. Several DNA vaccines based on TgPLP1, either mono, in combination with adjuvant, or multivalent, could elicit strong specific protective humoral and cellular immune responses against toxoplasmosis, notably prolong the survival time of mice infected with acute infection with *T. gondii*, and reduce the cysts number of mice brain during chronic *T. gondii* infection (10, 13). Furthermore, TgPLP1 is linked to promote *T. gondii* dissemination throughout the body by rapidly compromising the integrity of membranes encasing the parasite (14, 15).

So far, there is no effective and safe vaccine to prevent human toxoplasmosis (16). Researches are being done on developing safe and effective vaccines for humans to control toxoplasmosis, including inactivated vaccines, live attenuated vaccines, DNA vaccines, and subunit vaccines over past decades (16). To date, Toxovax is the only licensed vaccine against *Toxoplasma* (17). However, the use of this vaccine is not feasible for humans, since this vaccine format may revert to virulence. Considering the similarity of MACPF domain between TgPLP2 and TgPLP1, it is reasonable to suggest that TgPLP2 is a promising vaccine candidate. Therefore, in this study, we sought to investigate the immune protective efficacy of TgPLP2 in mice models of *T. gondii* infection. Specific anti-TgPLP2 antibody neutralization tests and passive and active immunization assays conducted in Bagg albino/c (BALB/c) mice suggested that TgPLP2 could provide partial protective for mice. The data from this study will lay a foundation for the development of a new type of vaccine that is cheaper, safer, and more efficient and also provide a novel idea for toxoplasmosis control strategies. This is of prominent relevance regarding *T. gondii* vaccine development as well as animal-based foods hygiene.

## Materials and Methods

### Ethical Statements

All animal experiments were reviewed and approved by the Ethics Committee of Xinxiang Medical University (No. 20170305).

### Animals and Parasites

Six-week-old female BALB/c mice and Sprague Dawley (SD) rats (body weight ~150 g) were purchased from Beijing Vital River Laboratory Animal Technology Co., Ltd. (Beijing, China), and maintained under specific pathogen-free standard conditions. *T. gondii* RH strain (type I) and PRU strain (type II) was provided by the Department of Human Parasitology of Xinxiang Medical University in Henan, China. The purified RH tachyzoites and PRU cysts were harvested as described previously (18).

### Preparation of *T. gondii* Soluble Antigens

*T. gondii* soluble antigen (sTAg) was prepared as described in previous studies (19). Briefly, the tachyzoites of *T. gondii* were obtained from peritoneal fluid of the BALB/c mice infected experimentally with RH strain (3 days Post-infection). Debris and host cells were further removed by using 27-gauge needles and 5-μm filter membranes. After that, tachyzoites were washed and re-suspended in phosphate-buffered saline (PBS buffer) (pH 7.4) then disrupted by sonication on ice. Finally, sTAg was stocked at −70°C for further use.

### Recombinant TgPLP2 Production and Generation of Polyclonal Antibodies Specific for Recombinant TgPLP2

According to the TgPLP2 (Accession No. TGGT1_272430) open reading frame from the ToxoDB (http://toxodb.org/toxo/), the MACPF domain of TgPLP2 (219–573aa) predicted by uniport (http://uniport.org/) was successively amplified and then constructed to the expression vector pET-30a. Software Primer Premier 5.0 was used to design the primer for TgPLP2 [primer -F: CGCcatatgATGCCTGAGAGCATGTACACGCGAGCCGT(*Nde* I); primer -R: CCC*aagctt*TCACTCAAGAGACTGAACGTCCATCGCGGA(*Hind* III)]. The recombinant protein was purified, and the endotoxin was removed using His Bind® Resin Chromatography kit (Merck, Darmstadt, Germany) and Detoxi-Gel Affinity Pak Prepacked columns (Pierce, Rockford, USA) as previously described (20). The purified proteins were stocked at −70°C for later use.

The SD rats were used to prepare polyclonal antibodies (PcAb) specific for recombinant TgPLP2 (rTgPLP2) as previously described (21). Subcutaneous immunizations were performed in duplicates for a total of four times. Briefly, 0.2 mg of purified rTgPLP2 protein formulated with Freund's complete adjuvant (Sigma-Aldrich, St Louis, MO, USA) (1:1) was injected into rats. The second injection was given to the rats after a 2-week interval, with 0.2 mg of purified rTgPLP2 protein mixed with Freund's incomplete adjuvant (Sigma-Aldrich) (1:1 in volume). At 1-week intervals, the remaining two injections were given to the immunized rats. One week after the last injection, the sera containing specific antibodies were collected and purified using octanoic acid-ammonium sulfate and stored at −30°C for further usage. Recognition of rTgPLP2 by purified antibodies was determined in Western blot (Supplementary Figure S1A). The specific antibody titer was analyzed by ELISA (1: 10^4^ in rats' serum, Supplementary Figure S1B).

### Immunofluorescence Analysis

Confirmation of TgPLP2 expression and location in tachyzoites was performed by an immunofluorescence assay (IFA) as previously described (22). Briefly, freshly isolated *T. gondii* tachyzoites were fixed with 4% paraformaldehyde on a poly-L-lysine-treated glass slide and then permeabilized using PBS containing 1% TritonX-100 for 10 min. The tachyzoites were washed four times for 5 min each in PBST containing 4% (w/v) bovine serum albumin (BSA). Then the normal rat IgG (control) and the rat anti-rTgPLP2 PcAb (1:100 dilution) was added separately and incubated overnight at 4°C followed by staining with the goat anti-rat IgG coupled to Cy3 (1:100 dilution, Beyotime, Shanghai, China) for 40 min. 4′,6-Diamidino-2-phenylindole (DAPI, Beyotime, Shanghai, China) was used for nuclear staining. Ultimately, the binding was determined by checking the staining patterns with a ×100 oil objective lens on fluorescence microscope (Nikon, Beijing, China).

### Immunogenicity Analysis of Natural and Recombinant TgPLP2

This experimental method referred to the previous research (22). Separated sTAg or rTgPLP2 proteins in sodium dodecyl sulfate polyacrylamide gel electrophoresis (SDS-PAGE) gel were transferred onto nitrocellulose membrane (Millipore, Shanghai, China), respectively. The membranes were blocked with 5% (w/v) skim milk/PBS-0.5% Tween 20 (PBST) and then individually incubated with specific anti-TgPLP2 rat sera or serum from *T. gondii*-infected mice (1:100 dilution) for 1 h at 37°C. Next, each membrane was washed thrice with PBST for 5 min, and goat anti-rat IgG-horseradish peroxidase (HRP) or goat anti-mouse IgG-HRP (Sigma, Shanghai, China) was applied as secondary antibodies, respectively. Finally, an enhanced chemiluminescence (ECL) kit (Vazyme, Nanjing, China) was used to visualize the bands on the basis of operating manuals.

### Specific Anti-TgPLP2 Antibody Neutralization Tests

Pretreated tachyzoite for mice challenge was performed as previously described (19). *T. gondii* tachyzoites were suspended in PBS/antibiotics buffer to a concentration of 4 × 10^7^/ml. Then, 100 μl tachyzoites were respectively mixed with various concentrations of rat anti-rTgPLP2 PcAb (100, 200, 500, or 1,000 μg/ml), rat normal IgG, or PBS alone in a 1:1 volumetric ratio and incubated under constant rocking at 37°C for 30 min. Following incubation, the parasites were harvested and resuspended in 1 ml of sterile PBS. Then 1 × 10^2^ RH strain pretreated tachyzoites were given intraperitoneally to mice (10 mice per group). *T. gondii* infection was defined as day 0, and the survival of mice was monitored every 12-h interval until all mice were dead.

### Passive Immunization of Mice Assays

The protection of passive immunization was evaluated by mice survival rate. The protocol for passive immunization assays was prepared with reference to Wang et al. (19). Mice (10 mice per group) intraperitoneally received different concentrations (100, 200, 500, or 1,000 μg/ml) of rat anti-rTgPLP2 PcAb (200 μl/mouse) twice a week for 2 weeks, and the controls were injected with rat normal IgG or PBS alone in the same manner, respectively. One week after the last injection, the injected mice were challenged intraperitoneally with 1 × 10^2^ RH strain tachyzoites. *T. gondii* infection was defined as day 0, and the survival of mice was monitored every 12-h interval until all mice were dead.

### Active Immunization of Mice Assays

Given that recombinant antigen is the most applicable form in production of vaccines and sero-diagnostic tools on a commercial basis (23), the rTgPLP2 was prepared for active immunization. Meanwhile, water-in-oil emulsion ISA-201 was chosen for this study due to its obvious advantages, including easy subcutaneous injection, high safety level, and fast absorption.

A total of 90 BALB/c mice were randomized into three groups (30/group); 20 μg of rTgPLP2 was injected subcutaneously into each mouse with an equal volume of ISA 201 (Seppic, France), and mice immunized with ISA 201 alone or PBS only were enrolled as controls. All mice were vaccinated 3 times at 2-week intervals, and the first day of inoculation was considered as week 0. To determine antibodies evaluation and cytokine measurement, the blood of mice was taken on weeks 0 and 6 separately, and the collected sera were stored at −20°C for later use. Two weeks after the last vaccination, 10 immunized mice per group were challenged intraperitoneally with 1 × 10^2^ tachyzoites of RH *T. gondii* strain, and 10 additional mice were challenged intragastrically with 10 cysts of PRU *T. gondii*. The day of challenged with *T. gondii* RH tachyzoites was defined as day 0, and mice infected with RH strain tachyzoites were monitored every 12-h interval until all mice were dead. The survival of animals challenged with the PRU cysts was observed daily for up to 60 days Post-infection, and the brain of each mouse was removed and ground with 1 ml PBS. A total of 10 μl mixture of brain from each mouse was used to count cyst number with three replicates. Cyst images were captured by digital camera with Toupview 3.7 software, and diameters were measured by Motic image software. The ratio of the number or size reduction was calculated as given below: cysts number from PBS control mice – vaccinated mice/PBS control mice ×100%, and cysts diameter from PBS control mice – vaccinated mice/PBS control mice ×100%, respectively.

### Measurement of Antibody Responses in Serum by ELISA

As described previously, serum levels of anti-rTgPLP2 antibodies (total IgG, IgG1, and IgG2a) in all vaccinated mice were tested by ELISA with rTgPLP2 (24). Briefly, the ELISA plate was coated using 5 μg/ml rTgPLP2 for total IgG, IgG1, and IgG2a at 4°C overnight. Skimmed milk at a 5% concentration was used for blocking at 37°C for 1 h. Different mice sera (1:100) were added and incubated at 37°C for 1 h to detect IgG, IgG1, and IgG2a. Following washes with PBST, 1:8,000 dilutions of HRP-conjugated anti-mouse IgG, IgG1, or IgG2a were added and incubated for 1 h at 37°C. Plates were developed with 3,3,5,5-tetramethylbenzidine (TMB) for 20 min, and the reaction was terminated by using 2 M H_2_SO_4_. The absorbance at 450 nm was assayed using a microplate reader (MULTISKAN FC, Thermo scientific, Waltham, MA, United States).

### Cytokine Determination

To ascertain cytokine production levels, sera from each experimental group were obtained as described previously. IFN-γ, IL-12p70, IL-4, and IL-10 were measured using ready ELISA kits according to the manufacturer's instructions (Boster Systems, Wuhan, China). Cytokine concentrations were determined by reference to standard curves constructed with known amounts of mouse recombinant IFN-γ, IL-12p70, IL-4, and IL-10. Four-parameter logistic regression was used for curve fitting. The concentrations of four cytokines were presented as picograms per milliliter (pg/ml).

### Statistical Analysis

Graphpad Premier 7.0 software package (Graphpad Prism, San Diego, CA, USA) was used for statistical analysis. The differences of the data (e.g., antibody responses, cytokine level) between all the groups were compared by one-way ANOVA. Survival times for infected mice were estimated using the Kaplan–Meier method. Comparisons between groups were deemed significant if the *p*-value was smaller than 0.05. Statistical significance was set at ^*^p < 0.05, ^**^p < 0.01, ^***^p < 0.001; ns indicates Non-significance.

## Results

### Cloning, Expression, and Purification of TgPLP2

**Figure 1:**
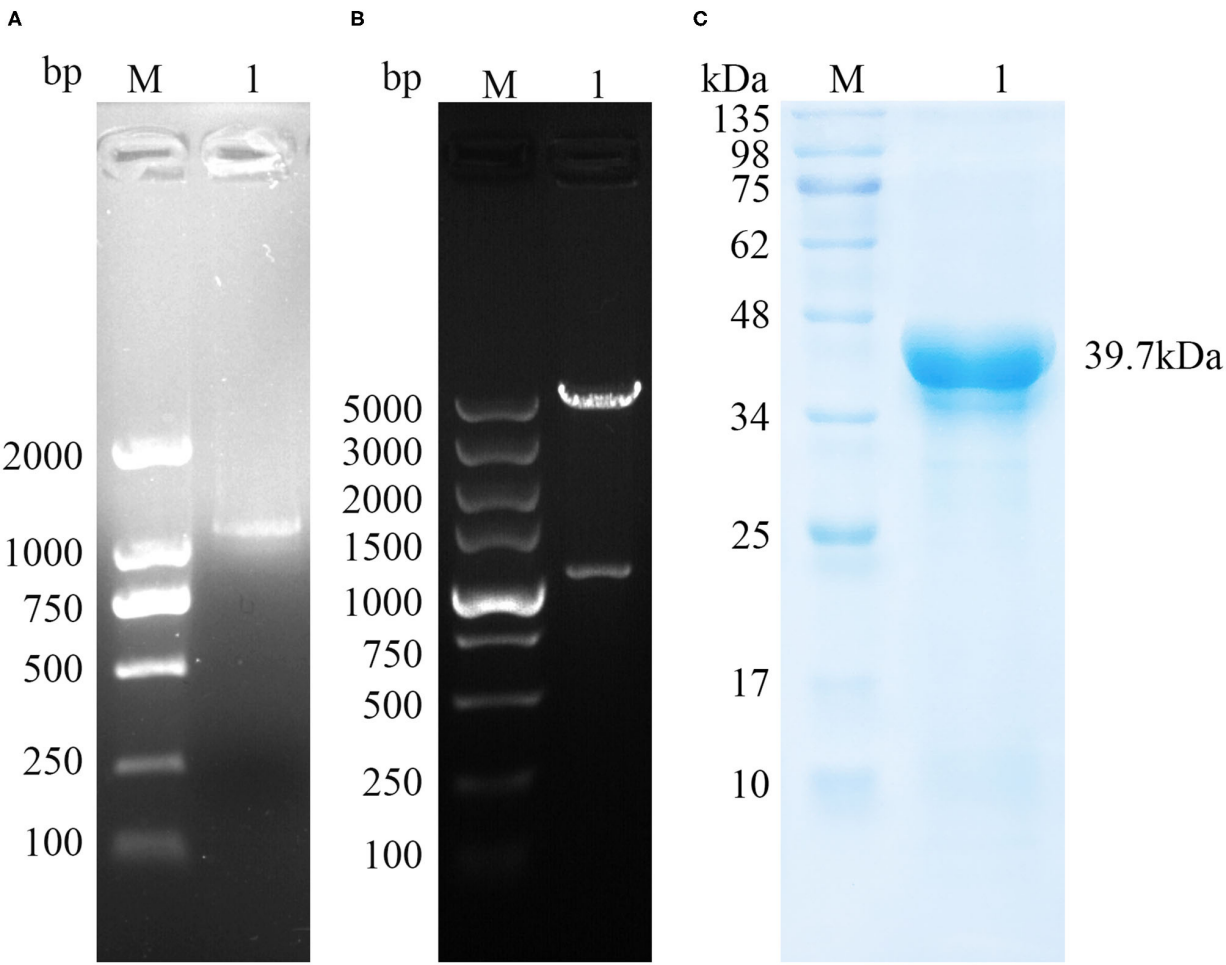
Gene cloning and protein purification of TgPLP2. **(A)** The amplification of TgPLP2 determined by agarose gel electrophoresis. **(B)** Double-enzyme digestion of pET30a-TgPLP2. **(C)** Purified rTgPLP2 was analyzed by SDS-PAGE gel and visualized by Coomassie blue staining.

The amplified TgPLP2 appeared as a single band in 1% agarose gel (Figure 1A). The target band was then cloned into pET-30a expression vector, confirmed by restriction digestion (Figure 1B) and sequenced. To permit expression of gene encoding the TgPLP2 protein in *T. gondii*, recombinant plasmid pET-30a-TgPLP2 was introduced into the BL21(DE3) strain to yield rTgPLP2. Then rTgPLP2 was purified by nickel column affinity chromatography. Target products rTgPLP2 was observed as a single band at 39.7 kDa level in SDS-PAGE (12%) gel (Figure 1C).

### Expression and Location of TgPLP2 in Tachyzoites of *T. gondii*

**Figure 2:**
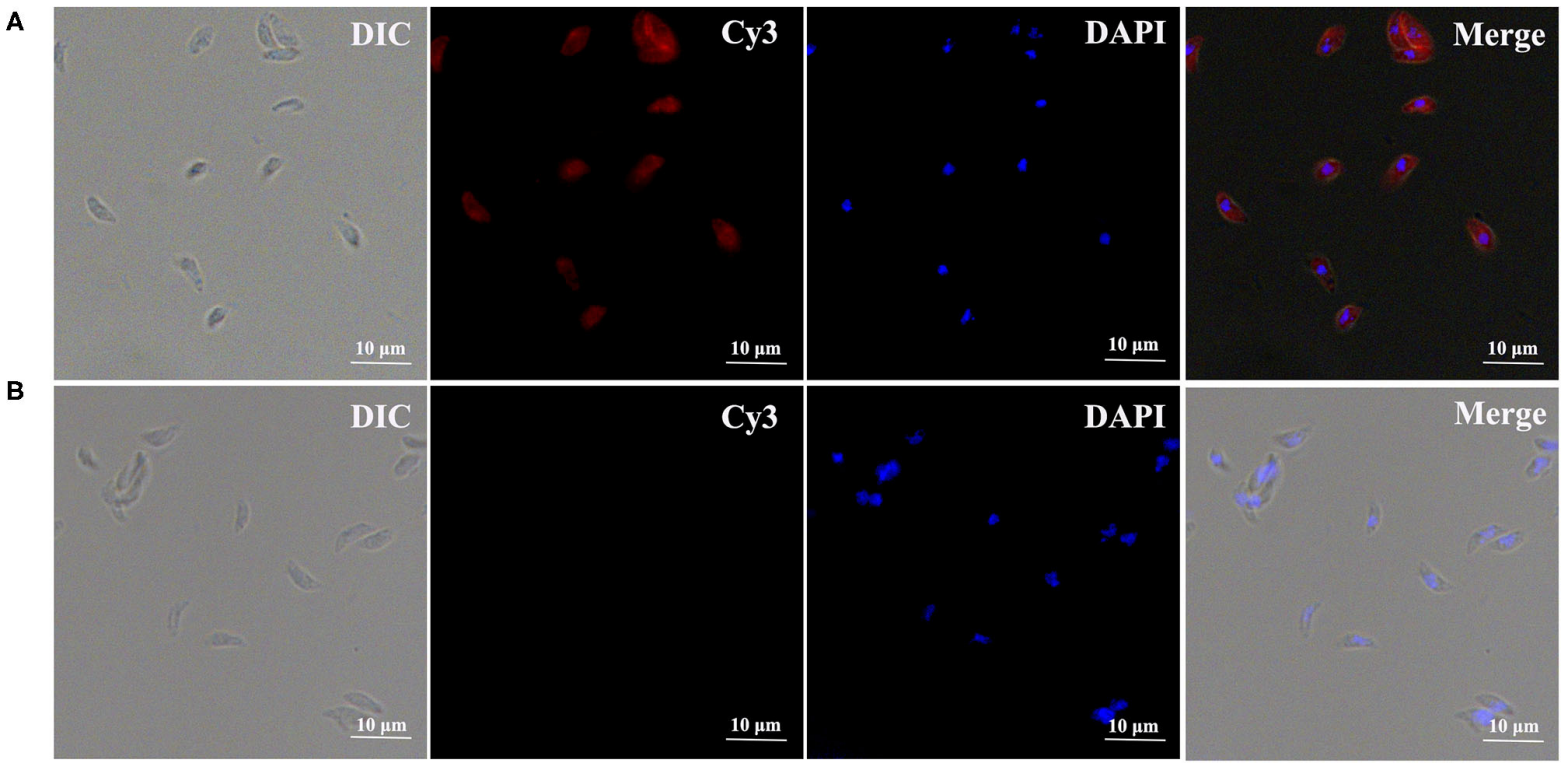
Expression and distribution of TgPLP2 in *T. gondii* tachyzoites. DAPI-stained nuclei and Cy3-labeled TgPLP2 appeared blue and red, respectively. **(A)** Anti-rTgPLP2 PcAb was used as primary antibody. **(B)** As control, rat normal IgG was used as primary antibody.

To investigate the localization of TgPLP2 in tachyzoites, immunofluorescence staining was used. The results indicated that TgPLP2 was expressed in tachyzoites and, for the most part, localized to the surface (Figure 2).

### Immunogenicity of TgPLP2

**Figure 3:**
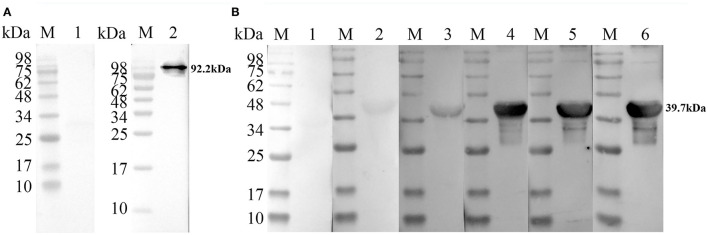
Recognition of native and recombinant TgPLP2 proteins. **(A)** Anti-rTgPLP2 PcAb recognized native TgPLP2 protein form sTAg. M, protein molecular mass standards; Line 1, rat normal IgG was used as control; Line 2, sTAg was transferred to nitrocellulose membrane, and anti-rTgPLP2 PcAb was used as primary antibody. **(B)** Recognition of rTgPLP2 by toxoplasmosis mice sera. M, protein molecular mass standards; Line 1, mouse normal IgG was used as control; Line 2, serum collected from *T. gondii* PRU strain-infected mice at 3 weeks Post-infection (wk p.i.) was used as primary antibody; Line 3, serum collected from *T. gondii* PRU strain-infected mice at 6 wk p.i. was used as primary antibody; Line 4, serum collected from *T. gondii* PRU strain-infected mice at 9 wk p.i. was used as primary antibody; Line 5, serum collected from *T. gondii* PRU strain-infected mice at 30 wk p.i. was used as primary antibody; Line 6, serum collected from *T. gondii* PRU strain-infected mice at 49 wk p.i. was used as primary antibody.

To evaluate the feasibility of TgPLP2 as a putative vaccine candidate, we primarily analyzed its immunogenicity. For detection of native TgPLP2, a band was visible at 92.2 kDa, indicating the specific antibodies could recognize the native parasite protein. The normal rat IgG was used as control where no band was seen (Figure 3A). As shown in Figure 3B, immunoblotting of rTgPLP2 incubated with sera from different *T. gondii*-infected mice strains showed clear bands around 39.7 kDa, whereas there was no band in the negative control. Immunoblotting showed that the native TgPLP2 from sTAg could be recognized by the specific rat serum against rTgPLP2. More than that, the rTgPLP2 could also be identified by the mice sera collected at different times after *T. gondii* infection, which illustrates the persistent expression of TgPLP2 in tachyzoites.

### Rat Anti-rTgPLP2 PcAb Obviously Curbed Tachyzoites Propagation

To measure the tachyzoites killing activity of anti-rTgPLP2 PcAb, the pretreated *T. gondii* tachyzoites were injected into BALB/c mice. As presented in Table 1, the survival time of mice in PBS group and rat normal IgG group was 8.78 ± 1.44 and 9.61 ± 1.9 days, respectively. There was no significant difference in survival time between anti-rTgPLP2 PcAb treatment groups and PBS control group (*p* > 0.05). The survival rate was 40% in mice treated with anti-rTgPLP2 PcAb (200 and 500 μg/ml), 20% in mice treated with anti-rTgPLP2 PcAb (1,000 μg/ml), and 0% in mice treated with anti-rTgPLP2 PcAb (100 μg/ml). The survival rate of mice in the PBS group and rat normal IgG group was 0%.

**Table 1:** Survival days of BALB/c mice challenged intraperitoneally with 10^2^ tachyzoites pretreated with anti-rTgPLP2 antibodies.

**Group**	**Survival days^**a**^**	***p*-value**	**Survival rate (%)**
PBS control	8.78 ± 1.44	–	0
Rat normal IgG control	9.61 ± 1.9	0.3366	0
Anti-rTgPLP2-A	9.4 ± 1.33	0.2326	0
Anti-rTgPLP2-B	8.83 ± 0.26	0.7581	40
Anti-rTgPLP2-C	9.08 ± 1.74	>0.9999	40
Anti-rTgPLP2-D	8.69 ± 0.7	>0.9999	20

*The repeated measures analysis of variance methods was employed for statistical analysis. Anti-rTgPLP2-A, mice were infected with rat anti-rTgPLP2 PcAb (100 μg/ml) pretreated tachyzoites; anti-rTgPLP2-B, mice were infected with rat anti-rTgPLP2 PcAb (200 μg/ml) pretreated tachyzoites; anti-rTgPLP2-C, mice were infected with rat anti-rTgPLP2 PcAb (500 μg/ml) pretreated tachyzoites; anti-rTgPLP2-D, mice were infected with rat anti-rTgPLP2 PcAb (1,000 μg/ml) pretreated tachyzoites*.

a *All mice were monitored 20 days Post-infection. Data were presented as mean ± standard deviation*.

### Passive Immunization With Rat Anti-rTgPLP2 PcAb Significantly Increased the Survival of Mice

Passive immunization and challenge experiments were performed in mice to assess the protective capacity of rat anti-rTgPLP2 PcAb. As shown in Table 2, the survival time of mice infected with *T. gondii* tachyzoites in PBS group and rat normal IgG group was 6.9 ± 3.11 and 8.33 ± 2.02 days, respectively, and there was no significant difference between the two groups (*p* > 0.05). The survival time of mice immunized with anti-rTgPLP2 PcAb (200 μg/ml) was significantly longer than that of mice in PBS group (^*^p < 0.05). The survival time of anti-rTgPLP2 PcAb immunization group (100, 500, and 1,000 μg/ml) was longer than that of PBS group; no significant difference was observed (*p* > 0.05). However, the survival rate of mice in each group was 0%.

**Table 2:** Survival days of BALB/c mice in passive immunization assay.

**Group**	**Survival days^**a**^**	***p*-value**	**Survival rate (%)**
PBS control	6.9 ± 3.11	-	0
Rat normal IgG control	8.33 ± 2.02	0.2965	0
Anti-rTgPLP2-A	9.5 ± 3.04	0.1223	0
Anti-rTgPLP2-B	12.13 ± 1.6	0.0046^**^	0
Anti-rTgPLP2-C	7.167 ± 2.255	0.8884	0
Anti-rTgPLP2-D	6.4 ± 0.89	0.266	0

*Anti-rTgPLP2-A, mice were passively immunized with rat anti-rTgPLP2 PcAb (100 μg/ml); anti-rTgPLP2-B, mice were passively immunized with rat anti-rTgPLP2 PcAb (200 μg/ml); anti-rTgPLP2-C, mice were passively immunized with rat anti-rTgPLP2 PcAb (500 μg/ml); anti-rTgPLP2-D, mice were passively immunized with rat anti-rTgPLP2 PcAb (1,000 μg/ml)*.

a *All mice were observed at 12-h intervals until all mice were dead. Data were presented as mean ± standard deviation*.

** *p < 0.01 compared with PBS control*.

### Humoral Immunological Response to rTgPLP2 Immunization

**Figure 4:**
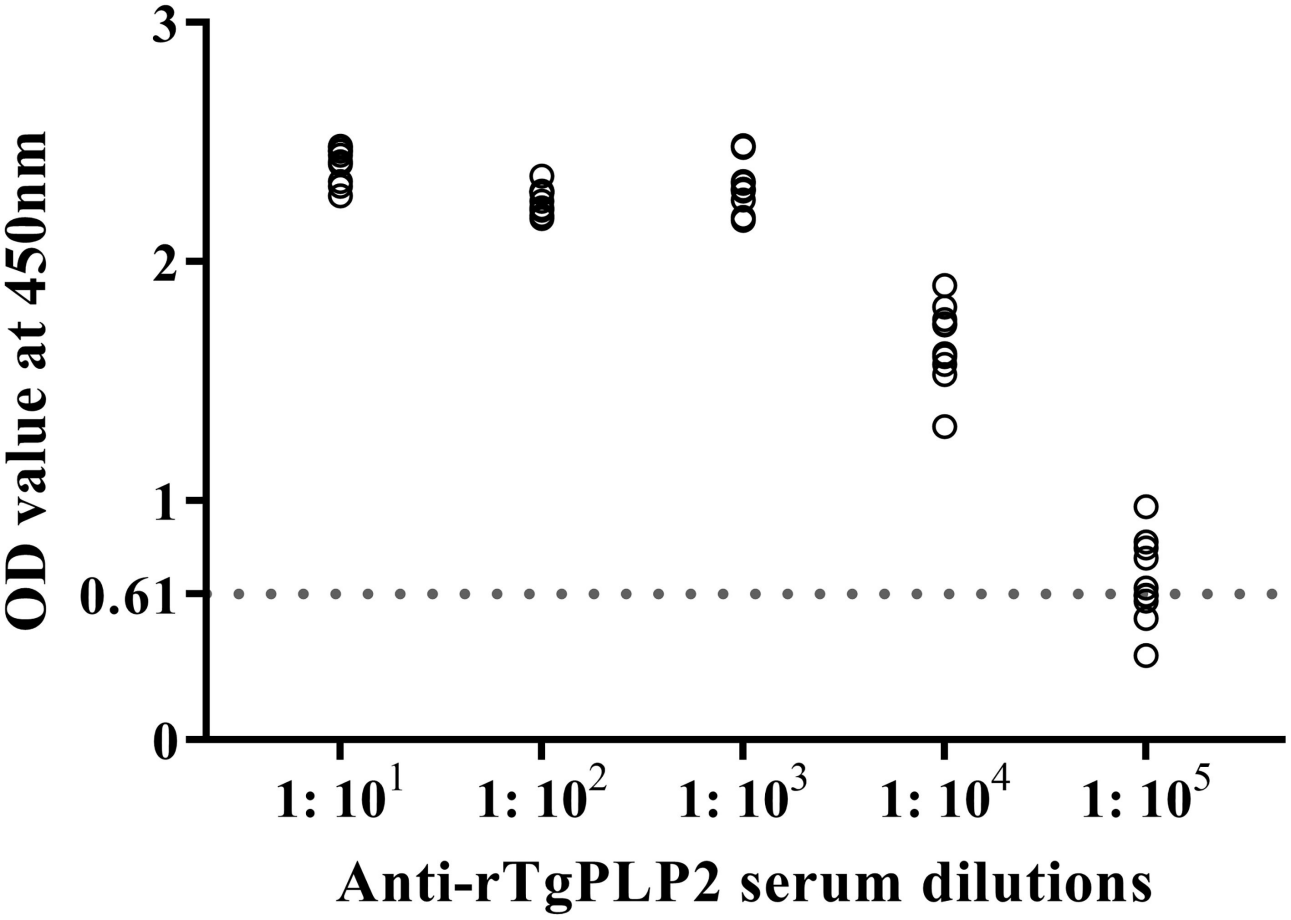
Serum titers of anti-rTgPLP2 IgG antibody measured by rTgPLP2-ELISA. Sera from 10 mice immunized with rTgPLP2 were taken for ELISA, and normal mice serum was used as negative control. The cut-off values (0.61) are shown with a dotted line.

**Figure 5:**
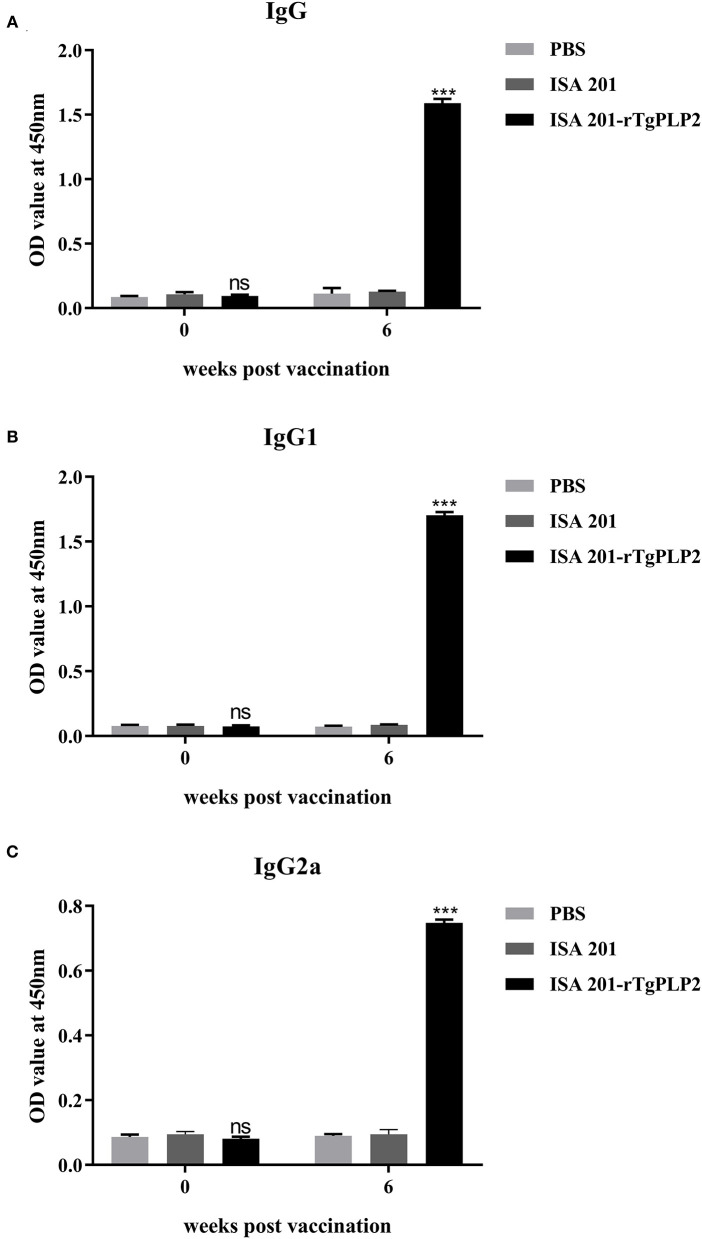
Specific anti-rTgPLP2 antibody responses in mice vaccinated with rTgPLP2. **(A)** Changes in specific IgG level at weeks 0 and 6 Post-vaccination. **(B)** Changes in specific IgG1 level at weeks 0 and 6 Post-vaccination. **(C)** Changes in specific IgG2a level at weeks 0 and 6 Post-vaccination. Data were expressed as mean ± SD for 10 mice per group. ^***^p < 0.001 and ns (Non-significant) vs. PBS or ISA 201 groups.

Sera from mice immunized three times and unimmunized controls were detected by ELISA against rTgPLP2 proteins. Anti-rTgPLP2 titers reached up to 1:10^4^ for vaccinated mice (Figure 4). Serum IgG levels induced by rTgPLP2 had a substantial increase compared with control groups (ISA 201 adjuvant and PBS control, ^***^p < 0.001) (Figure 5A). As indicated in Figures 5B,C, both IgG1 and IgG2a levels induced by rTgPLP2 showed considerable rise after inoculation (^***^p < 0.001), but IgG1 was the predominant subtype.

### Cytokine Response to rTgPLP2 Vaccination

**Figure 6:**
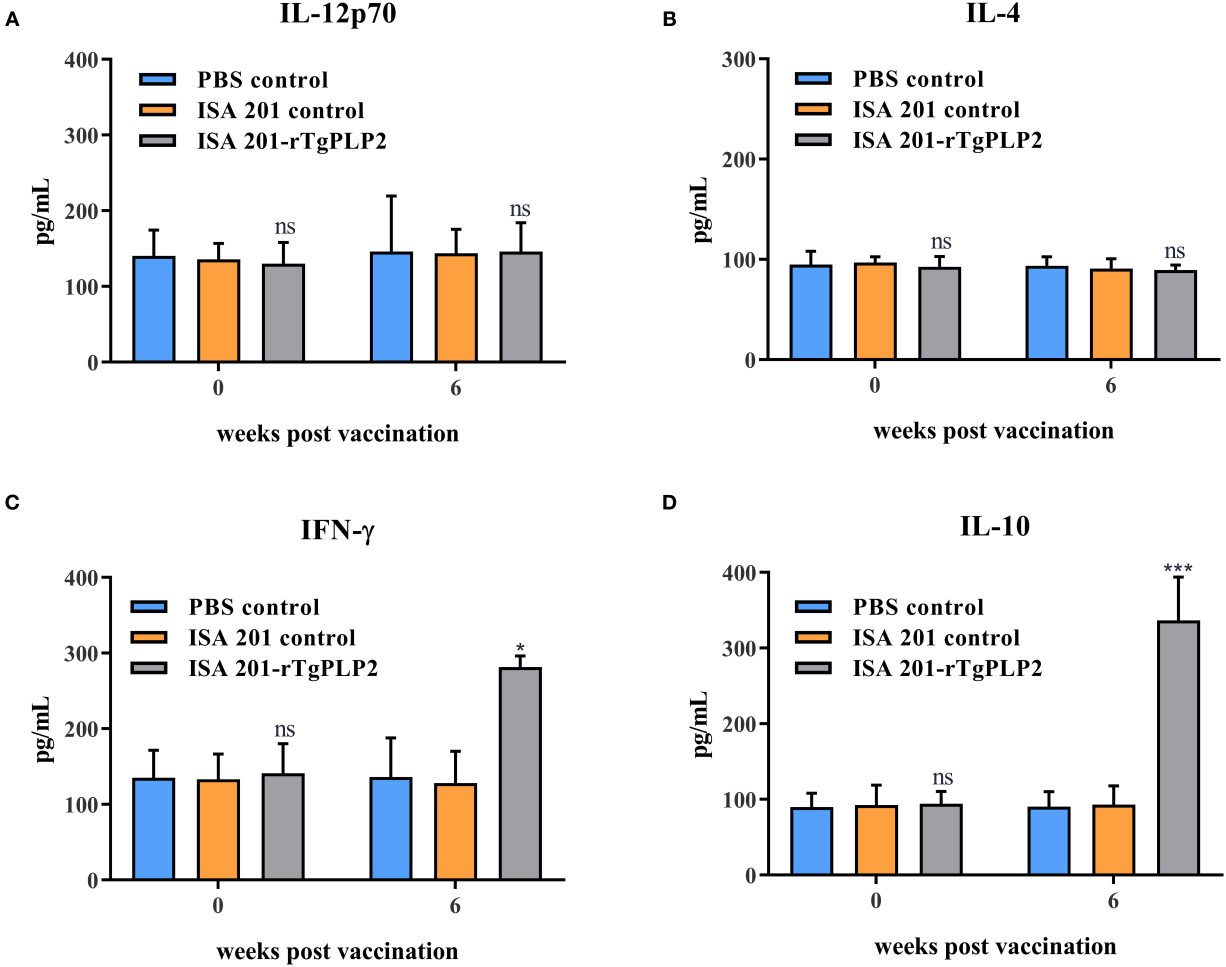
Cytokines IL-12p70 **(A)**, IL-4 **(B)**, IFN-γ **(C)**, and IL-10 **(D)** levels of mice vaccinated with rTgPLP2 at various times Post-vaccination. Data were expressed as mean ± SEM for 10 mice per group. *p < 0.05, ^***^p < 0.001 and ns (Non-significant) vs. PBS or ISA 201 groups.

To determine the cellular immune response, multiple cytokines (IL-12p70, IL-4, IFN-γ, and IL-10) in the serum were examined. Serum samples were collected at 0 and 6 weeks (2 weeks after the last immunization), respectively. The serum multiple cytokines levels of all groups prior to vaccination showed insignificant differences (*p* > 0.05) (Figures 6A–D). At 6 weeks Post-vaccination the levels of IL-12p70 and IL-4 were not statistically different among the groups (*p* > 0.05) (Figures 6A,B), but only the levels of IFN-γ and IL-10 in ISA201-rTgPLP2 immunized groups were significantly higher (*p* < 0.05 and *p* < 0.0001, respectively) than the control groups (Figures 6C,D).

### Evaluating the Vaccine Effectiveness of rTgPLP2 Against Challenge of *T. gondii*

**Figure 7:**
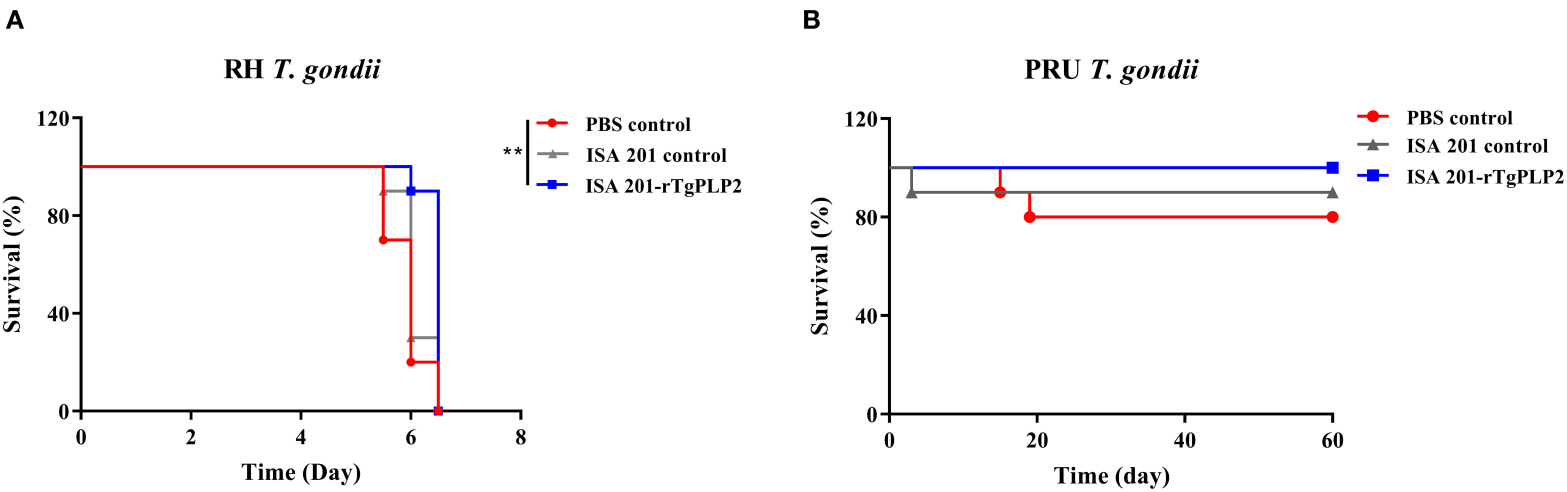
Survival curves of mice infected with *T. gondii* RH strain **(A)** and PRU strain **(B)**. The survival of PRU strain challenged mice were monitored for 60 days. **p < 0.01 compared with PBS controls.

Intraperitoneal inoculation of 10^2^ RH strain tachyzoites induced death of the mice within 7 days after the challenge (Figure 7A). There was no significant difference in survival time between the two control groups (PBS and ISA 201, *p* > 0.05). The survival time of mice immunized with ISA 201-rTgPLP2 was significantly longer than that of PBS group (*p* < 0.01).

**Figure 8:**
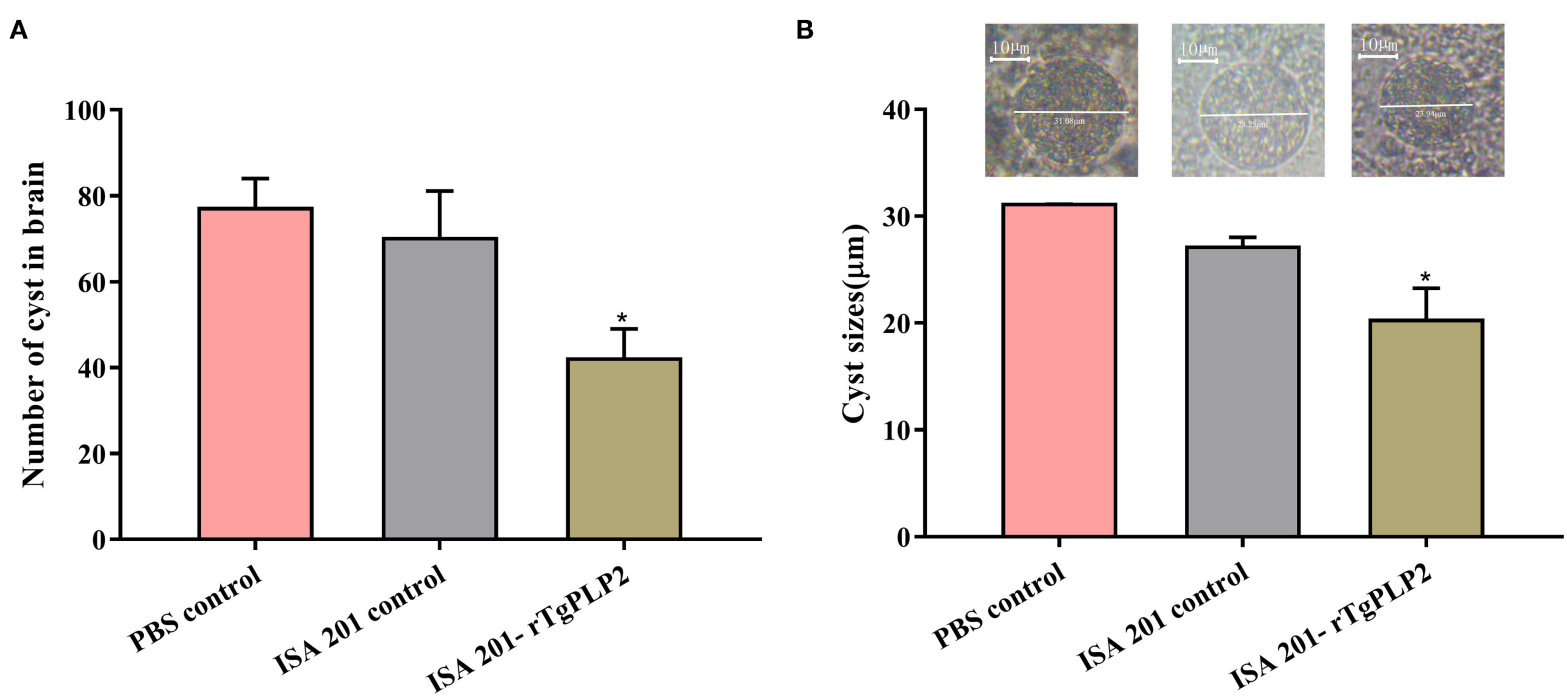
Number **(A)** and size **(B)** of brain cysts in the mice infected with *T. gondii* PRU strain. Data were expressed as mean ± SEM. ^*^p < 0.05 compared with PBS controls.

After 2 months of observation, 10 of 10 mice infected orally with 10 cysts of PRU strain in ISA 201-rTgPLP2 immunized group survived as indicated in Figure 7B. However, one animal in the ISA 201 group died, while two died in the PBS group throughout the experiment. Brain cyst burdens in ISA 201-rTgPLP2 immunized group were significantly reduced compared with PBS control groups (reduction by 45.45%, *p* < 0.05, Figure 8A and Table 3). Meanwhile, compared with PBS control groups, the brain cyst sizes of ISA 201-rTgPLP2 immunized group were statistically apparently smaller (*p* < 0.05, reduction by 24.89%, Figure 8B and Table 3).

**Table 3:** The number and size of PRU cysts in rTgPLP2 preimmunized mice brain.

**Groups**	**Cyst number (mean ±SD)**	**Cyst reduction rate (%)**	**Cyst size (μm) (mean ±SD)**	**Size reduction rate (%)**
PBS control	77 ± 15.7 a	0.00	31.1 ± 0.03 a	0.00
ISA 201 control	70 ± 24.8 ab	9.09	27.1 ± 1.59 ab	12.9
ISA 201-rTgPLP2	42 ± 15.7 b	45.5	20.3 ± 5.21 b	24.9

*Different letters in the same column represent significant difference, p < 0.05*.

## Discussion

Given the present and potential harms posed by *T. gondii*, the development of an effective human vaccine for toxoplasmosis is warranted. In this study, we evaluated the vaccine efficacy induced by rTgPLP2, and the results indicated that this recombinant form had favorable immunogenicity properties in mice, making it a promising vaccine candidate. Currently, apicomplexan PLPs (ApiPLPs) form *Plasmodium* and *Toxoplasma* have been evaluated as potential candidates since ApiPLPs are known to be related to host cell egress (10, 25), and this is also reflected in studies regarding protective immunity by TgPLP1. Thus, we assumed an antigen delivery system containing TgPLP2 could induce high-level protection against *T. gondii*. As anticipated, the monovalent formulation composed of rTgPLP2 and ISA-201 elicited persistent and excellent immune effects.

Surface proteins are usually presumed to be related to provide protection for the parasites against attacks by host immune systems (26). In the present study, we found TgPLP2 localized in surface of the tachyzoites, indicating that TgPLP2 appeared to be a good immunogen. Out of immunoblotting assays, compelling evidences suggested that TgPLP2 was immunogenic, which was not only because its antibody could recognize the recombinant protein and native parasite protein but also because it could be recognized by the infected mouse serum.

Passive immunization strategies have been utilized to treat or prevent infectious diseases (27). The transfer of mAbs, PcAbs against *T. gondii* proteins (20, 28, 29) or sera against *T. gondii* variant strains into infected mice promoted prolonged survival (30). Here, the contribution of anti-rTgPLP2 PcAb was proved by antibody neutralization tests; we noticed that mice challenged by antibody-pretreated tachyzoites had longer survival, especially when the antibodies concentrations were 200, 500, and 1,000 μg/ml. Moreover, mice passively immunized with anti-rTgPLP2 PcAb also showed a partial protection, although the immunized mice survival was only prolonged for about 4 days at the longest compared to controls. The above results underpinned the feasibility of PcAb-TgPLP2 as a passive immunotherapeutic.

As indicated in our immune response measurement studies, rTgPLP2 could strongly elicit antigen specific humoral response and cell-mediated immune responses. Assessment of specific IgG levels suggested that rTgPLP2 triggered an IgG1-dominant Th1/Th2-combined immune response, which was indicated by a significant rise of specific IgG1 and IgG2a. In addition, cytokine levels were detected to describe immunologic mechanisms induced by rTgPLP2. IL-12 and IFN-γ as Th1-type cytokines are crucially important to prevent *T. gondii* infection (31). Previous studies have suggested that IFN-γ could induce immunity against toxoplasmosis (32, 33). As was seen in this research, although IL-12 levels did not appear to increase, IFN-γ levels as expected exhibited a notably rise compared with controls, indicating rTgPLP2 vaccination might be beneficial in preventing *T. gondii* infection and enhancing cysts clearance within the brain. IL-10 was observed at a high level in mice serum; as a key immunoregulator, IL-10 inhibits the activity of Th1 cells to maintain the immune balance (34). Recent studies revealed that cytokine IL-10 played crucial roles in controlling the inflammatory response during acute *T. gondii* infection (35, 36). IL-4 is a Th2 cytokine and generally antagonizes IFN-γ function (37), which is consistent with high IFN-γ and low IL-4 concentrations presented in this study.

Both RH strain and PRU strain were selected in this work to evaluate protective efficacy of the rTgPLP2 for mice. Specifically, survival assays revealed a limited protection provided by rTgPLP2; the mice challenged with RH strain had about 0.5 day longer survival on average more than PBS controls. Moreover, it was noteworthy that immunization with rTgPLP2 could significantly reduce the size and number of brain cysts in PRU strain-infected mice. Obviously, the specific immunity and cytokines generated by rTgPLP2 might have driven these outcomes. Moreover, according to the results, we considered that rTgPLP2 vaccine possessed a better performance against chronic infection stage. However, only two different strains were evaluated in the present study; considering that different *T. gondii* strains had various levels of virulence, whether rTgPLP2 could provide effective protection against parasites needs further investigation.

In conclusion, our study suggested that rTgPLP2 has the potential to be used as a vaccine. Its subcutaneous immunization can activate a mixed humoral and cellular immune response, thereby protecting the hosts against *T. gondii* infection. Although rTgPLP2 possesses several features that can be explored as vaccine candidate from mice models, further researches are still needed to validate the protective effects in different strains infection.

## Data Availability Statement

The original contributions presented in the study are included in the article/Supplementary Material, further inquiries can be directed to the corresponding authors.

## Ethics Statement

The animal study was reviewed and approved by Ethics Committee of Xinxiang Medical University (Approval Number 20170305).

## Author Contributions

XL, SW, and XT: conception or design of this study. XM, ZZ, and XL: direction and supervision. XT: data analysis and drafting this article. XT, HS, MW, GW, and TX: material acquisition. All authors read and approved the final manuscript.

## Funding

The current work received support from the Science and Technology Planning Project of Henan Province (Nos. 212102310749 and 222102310557), the Key Scientific Research Projects of Colleges and Universities in Henan Province (No. 22A310004), the Training Plan for Young Backbone Teachers in Universities of Henan Province (No. 2021GGJS101), the Doctoral Scientific Research Activation Foundation of Xinxiang Medical University (No. XYBSKYZZ202139), and the Scientific Research Cultivation Project of School of Basic Medical Sciences in Xinxiang Medical University (No. JCYXYKY202103).

## Conflict of Interest

The authors declare that the research was conducted in the absence of any commercial or financial relationships that could be construed as a potential conflict of interest.

## Publisher's Note

All claims expressed in this article are solely those of the authors and do not necessarily represent those of their affiliated organizations, or those of the publisher, the editors and the reviewers. Any product that may be evaluated in this article, or claim that may be made by its manufacturer, is not guaranteed or endorsed by the publisher.

## Supplementary Material

Supplementary Figure S1 Immunoblotting analysis of specific antibody anti-rTgPLP2 **(A)**. Diaminobenzidine (DAB) substrate was used for band visualization. M showed standard protein ladder; Line 1: Normal mice sera were utilized as negative controls; Line 2: Anti-rTgPLP2 rat serum was used as primary antibody and a clear band about 39.7 kDa was observed. ELISA analysis of serum anti-rTgPLP2 antibody titers **(B)**. Normal mice sera were utilized as negative controls. The cut-off values (0.03) are shown with a dotted line.

Supplementary Figure S2 Original picture of Figure 1C.

Supplementary Figure S3 Original picture of Supplementary Figure S1.

Supplementary Figure S4 Original picture of Figure 3A Line 1.

Supplementary Figure S5 Original picture of Figure 3A Line 2.

Supplementary Figure S6 Original picture of Figure 3B.

Supplementary Figure S7 Original picture of Figure 1B.

Supplementary Figure S8 Original picture of Figure 1A.
