# Supplementary figures and images for: A Novel Vaccine Candidate: Recombinant Toxoplasma gondii Perforin-Like Protein 2 Stimulates Partial Protective Immunity Against Toxoplasmosis
Source: Front Vet Sci. 2022 Feb 15;8:802250. doi: 10.3389/fvets.2021.802250 (PMC8890382; doi:10.3389/fvets.2021.802250)

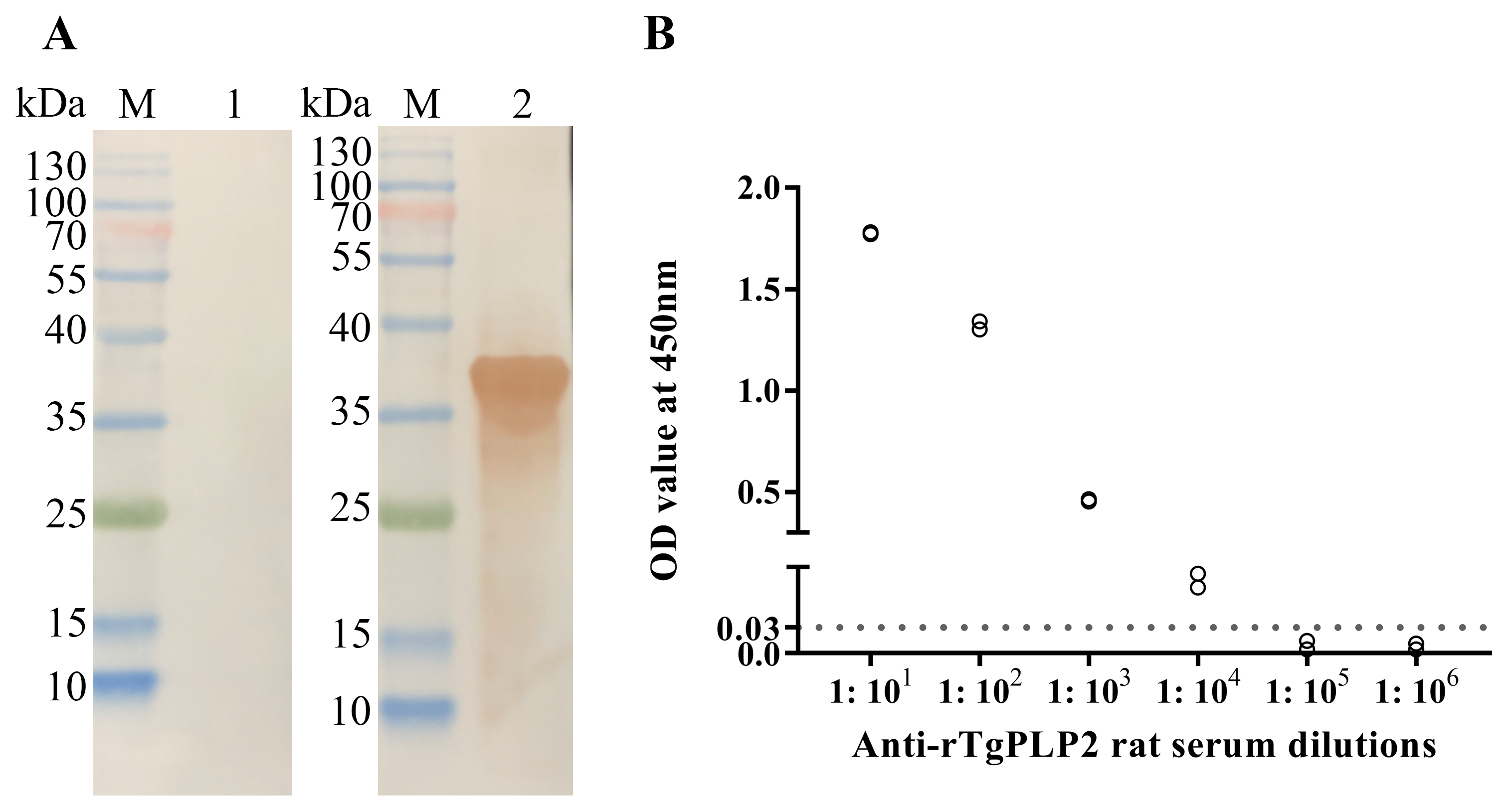

Supplement: Supplementary Figure S1 — Immunoblotting analysis of specific antibody anti-rTgPLP2 (A). Diaminobenzidine (DAB) substrate was used for band visualization. M showed standard protein ladder; Line 1: Normal mice sera were utilized as negative controls; Line 2: Anti-rTgPLP2 rat serum was used as primary antibody and a clear band about 39.7 kDa was observed. ELISA analysis of serum anti-rTgPLP2 antibody titers (B). Normal mice sera were utilized as negative controls. The cut-off values (0.03) are shown with a dotted line. [file Image_1.TIF]

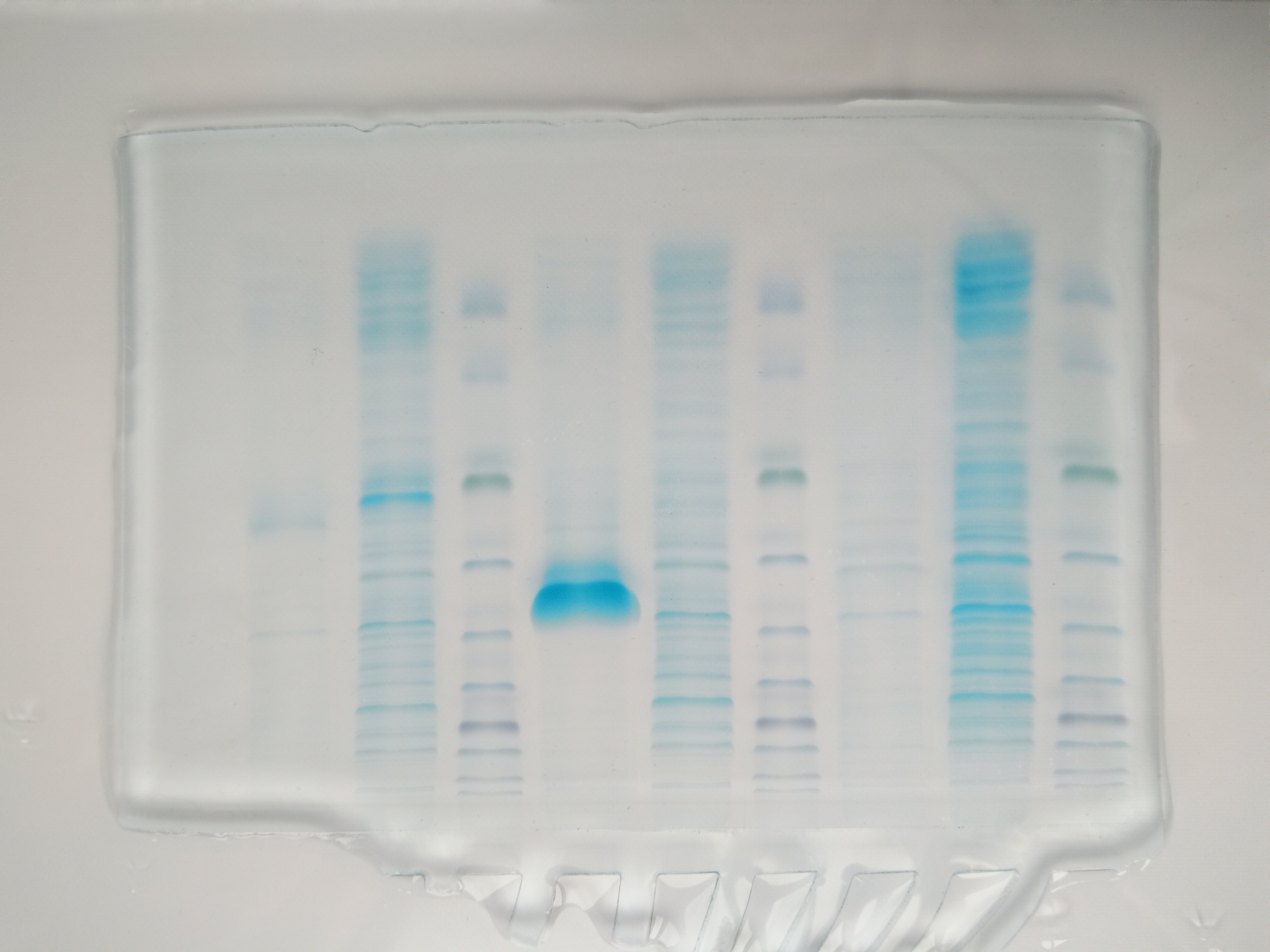

Supplement: Supplementary Figure S2 — Original picture of Figure 1C. [file Image_2.JPEG]

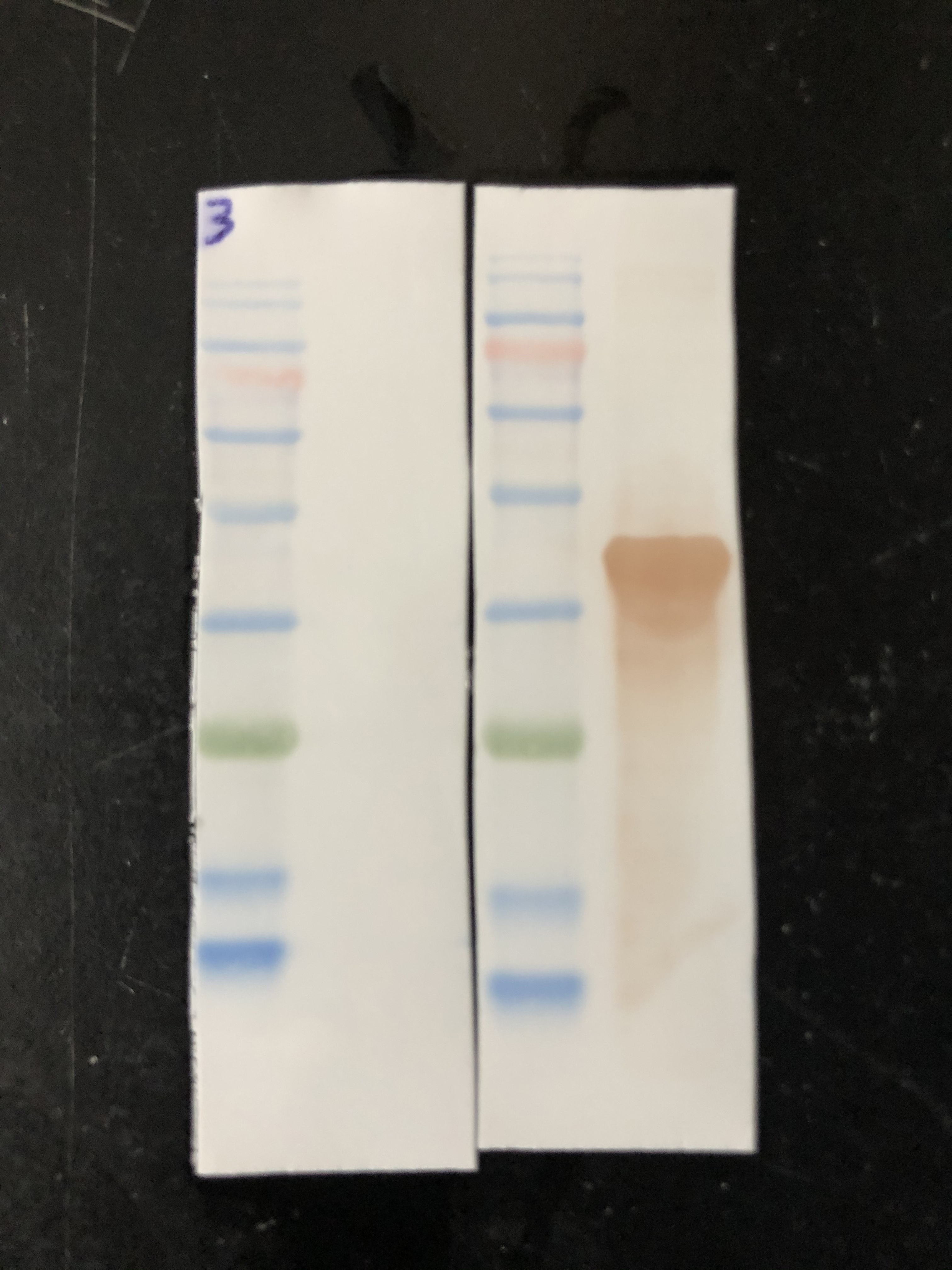

Supplement: Supplementary Figure S3 — Original picture of Supplementary Figure S1. [file Image_3.JPEG]

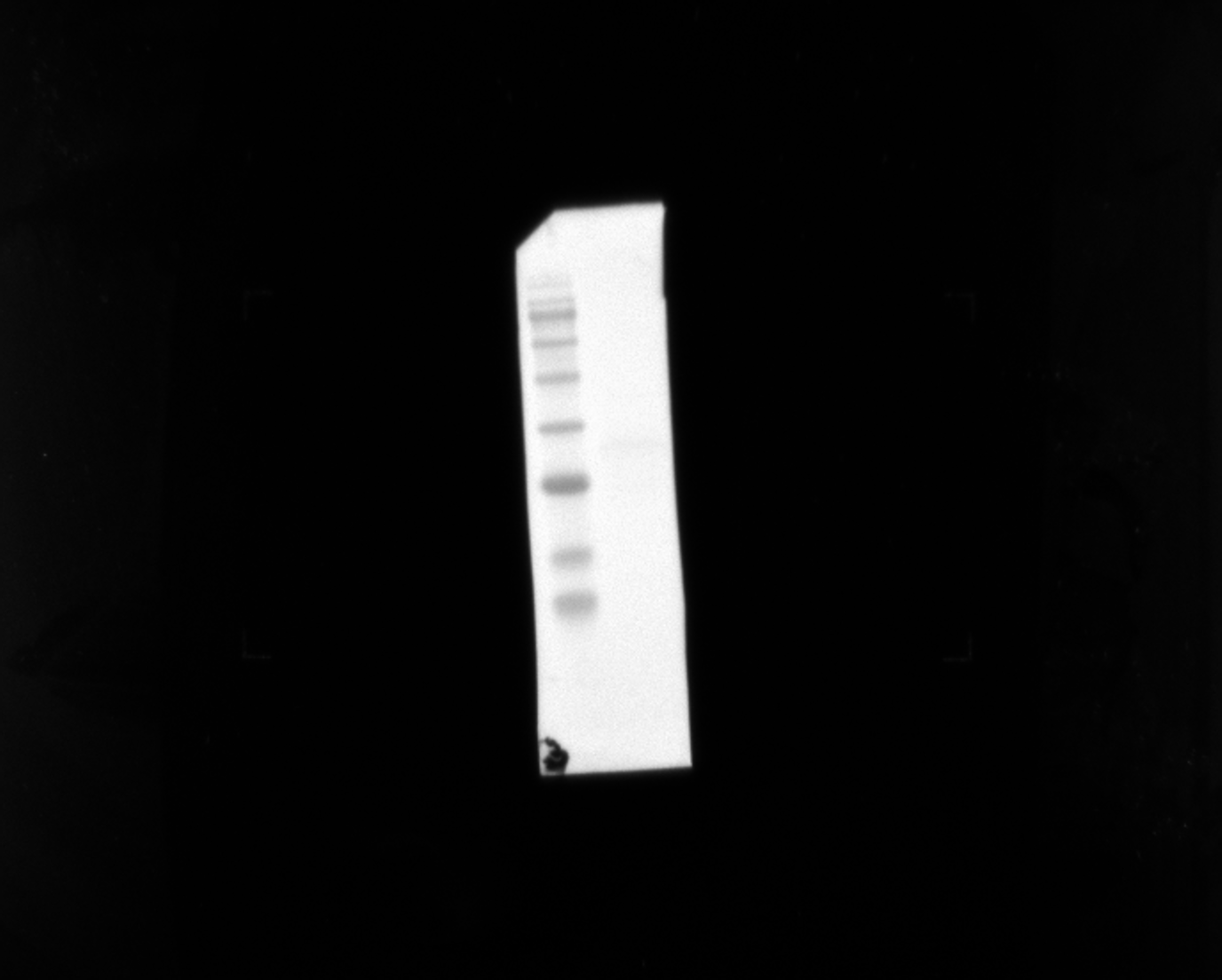

Supplement: Supplementary Figure S4 — Original picture of Figure 3A Line 1. [file Image_4.TIF]

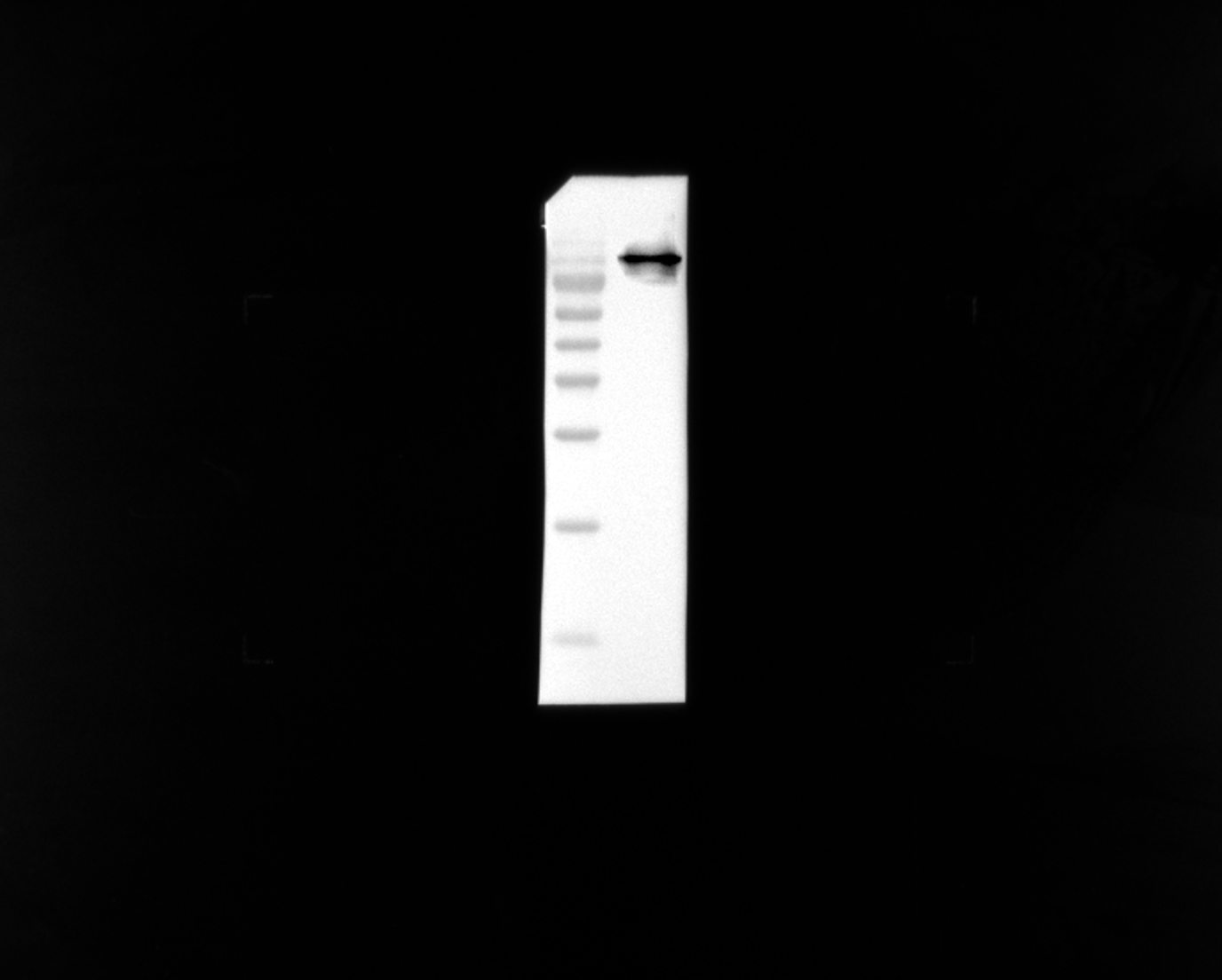

Supplement: Supplementary Figure S5 — Original picture of Figure 3A Line 2. [file Image_5.TIF]

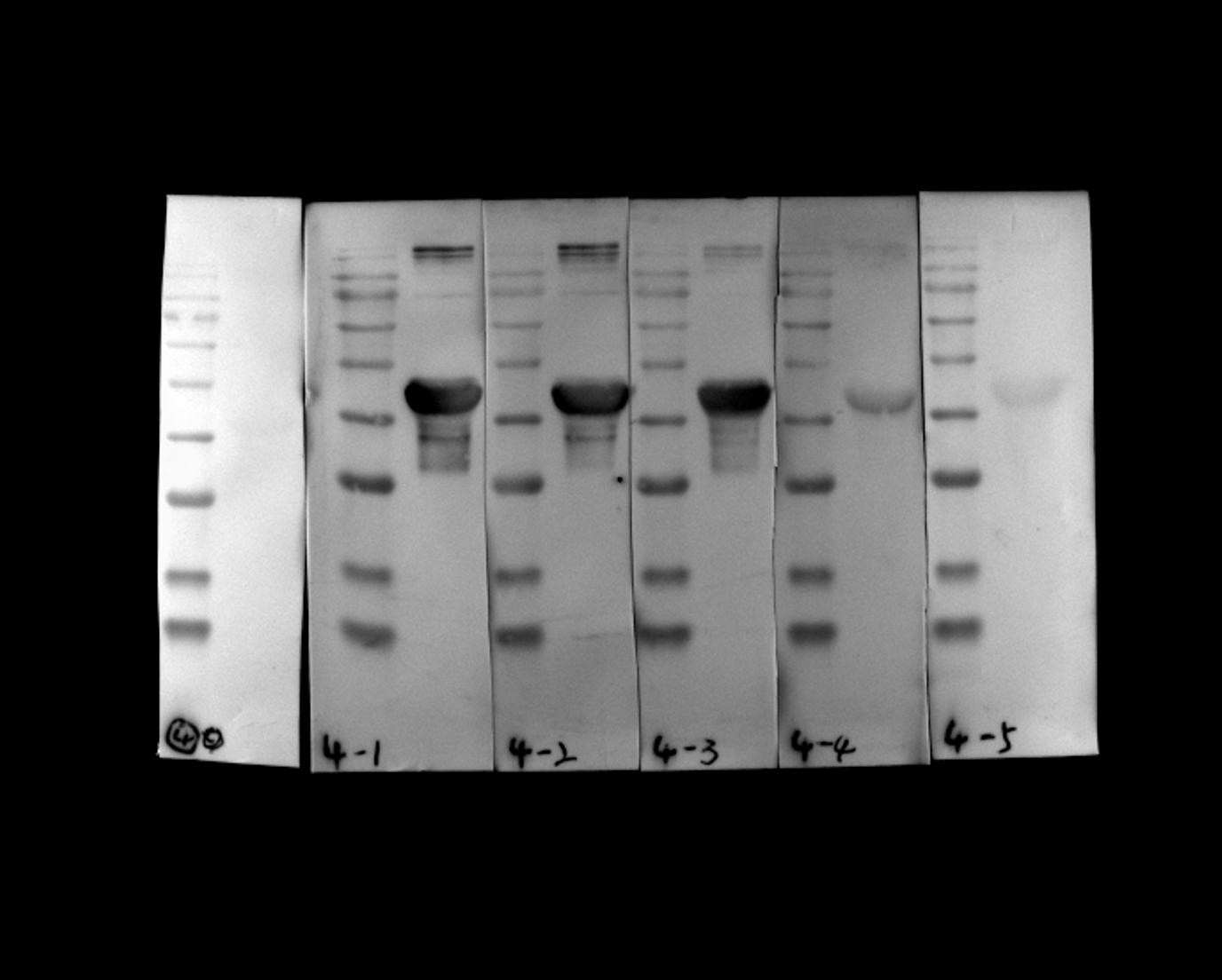

Supplement: Supplementary Figure S6 — Original picture of Figure 3B. [file Image_6.TIF]

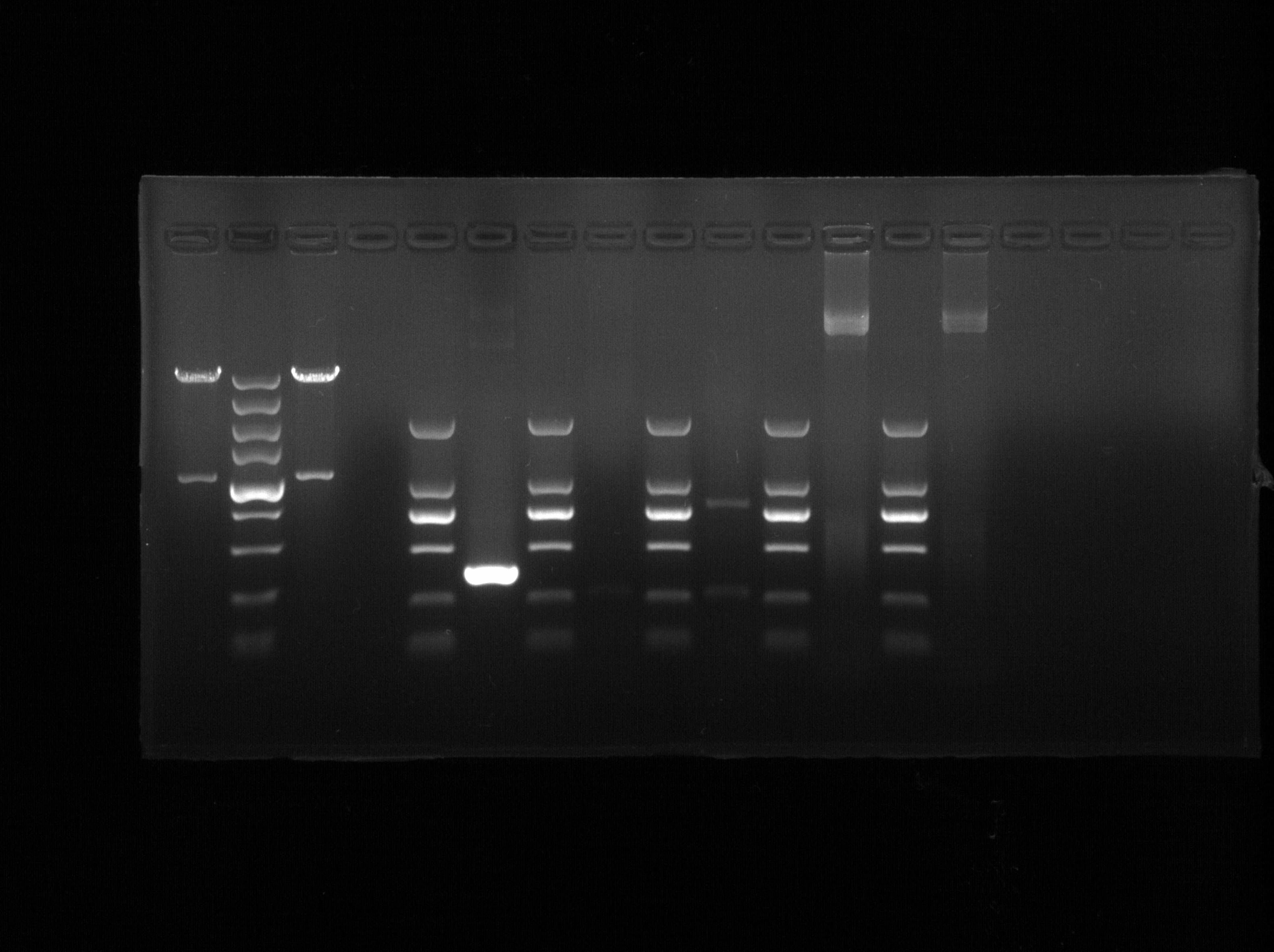

Supplement: Supplementary Figure S7 — Original picture of Figure 1B. [file Image_7.TIF]

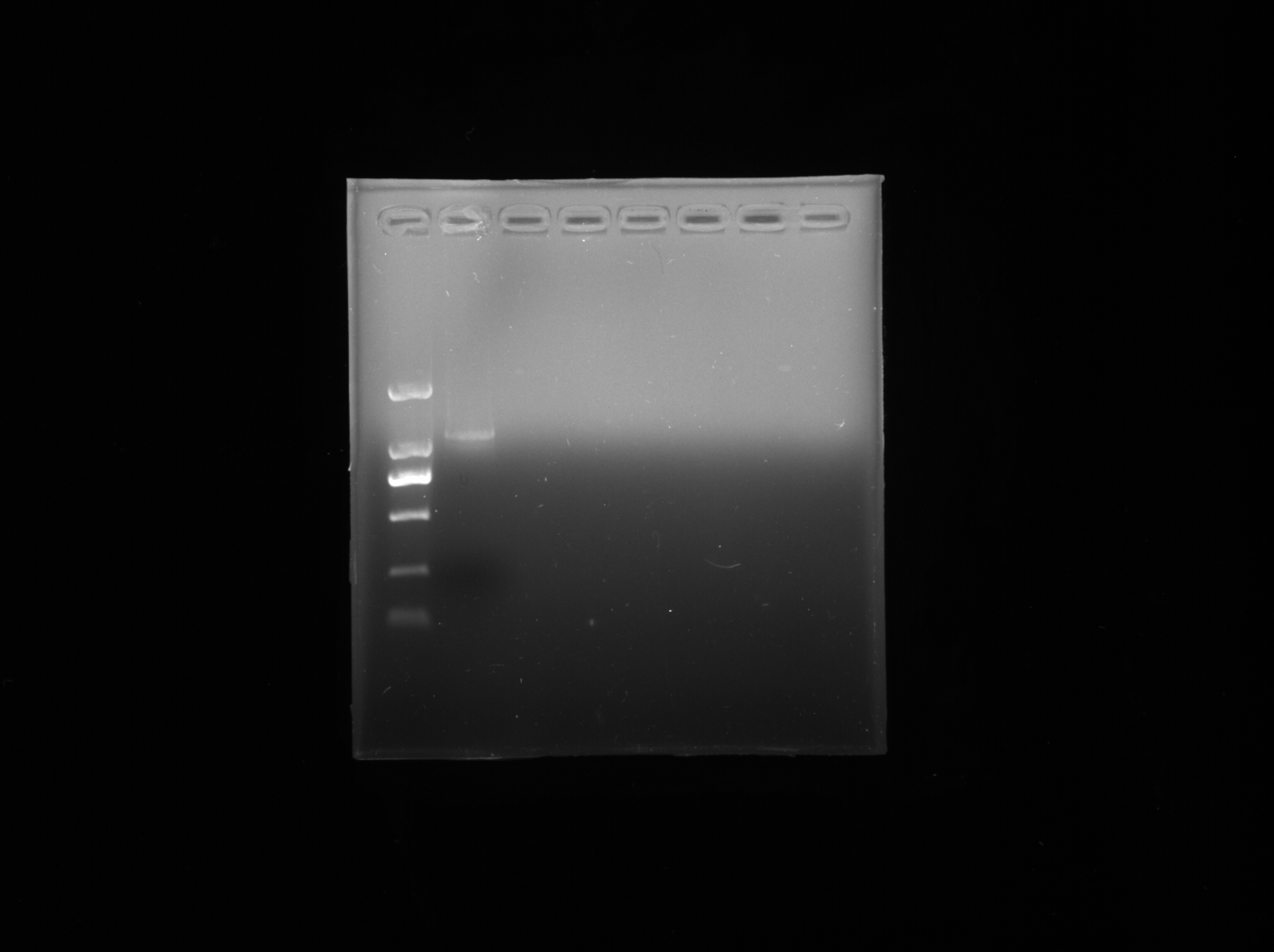

Supplement: Supplementary Figure S8 — Original picture of Figure 1A. [file Image_8.TIF]
